# ANOCA patients with and without coronary vasomotor dysfunction present with limited electrocardiographic remodeling

**DOI:** 10.1016/j.ijcha.2024.101347

**Published:** 2024-01-31

**Authors:** Diantha J.M. Schipaanboord, Tijn P.J. Jansen, Caïa Crooijmans, N. Charlotte Onland-Moret, Suzette E. Elias-Smale, Aukelien C. Dimitriu-Leen, Pim van der Harst, Tim P. van de Hoef, René van Es, Peter Damman, Hester M. den Ruijter, on behalf of the IMPRESS consortium

**Affiliations:** a Laboratory of Experimental Cardiology, University Medical Center Utrecht, Utrecht University, Utrecht, The Netherlands; b Department of Cardiology, Radboud University Medical Center, Nijmegen, The Netherlands; c Julius Center for Health Sciences and Primary Care, University Medical Center Utrecht, Utrecht University, Utrecht, The Netherlands; d Department of Cardiology, Division Heart and Lungs, University Medical Centre Utrecht, Utrecht University, Utrecht, The Netherlands

**Keywords:** Electrocardiography, Coronary vasomotor dysfunction, Coronary spasm, Coronary microvascular dysfunction, QTc interval

## Abstract

**Background:**

Coronary vasomotor dysfunction (CVDys) comprises coronary vasospasm (CVS) and/or coronary microvascular dysfunction (CMD) and is highly prevalent in patients with angina and non-obstructive coronary artery disease (ANOCA). Invasive coronary function testing (CFT) to diagnose CVDys is becoming more common, enabling pathophysiologic research of CVDys. This study aims to explore the electrophysiological characteristics of ANOCA patients with CVDys.

**Methods:**

We collected pre-procedural 12-lead electrocardiograms of ANOCA patients with CVS (n = 35), CMD (n = 24), CVS/CMD (n = 26) and patients without CVDys (CFT-, n = 23) who participated in the NL-CFT registry and underwent CFT. Heart axis and conduction times were compared between patients with CVS, CMD or CVS/CMD and patients without CVDys.

**Results:**

Heart axis, heart rate, PQ interval and QRS duration were comparable between the groups. A small prolongation of the QT-interval corrected with Bazett (QTcB) and Fridericia (QTcF) was observed in patients with CVDys compared to patients without CVDys (CVS vs CFT-: QTcB = 422 ± 18 vs 414 ± 18 ms (p = 0.14), QTcF = 410 ± 14 vs 406 ± 12 ms (p = 0.21); CMD vs CFT-: QTcB = 426 ± 17 vs 414 ± 18 ms (p = 0.03), QTcF = 413 ± 11 vs 406 ± 12 ms (p = 0.04); CVS/CMD vs CFT-: QTcB = 424 ± 17 vs 414 ± 18 ms (p = 0.05), QTcF = 414 ± 14 vs 406 ± 12 ms (p = 0.04)).

**Conclusions:**

Pre-procedural 12-lead electrocardiograms were comparable between patients with and without CVDys undergoing CFT except for a slightly longer QTc interval in patients with CVDys compared to patients without CVDys, suggesting limited cardiac remodeling in patients with CVDys.

## 1 Introduction

Patients with angina and non-obstructive coronary artery disease (ANOCA) are increasingly recognized to have a high prevalence of coronary vasomotor dysfunction (CVDys), which comprises coronary vasospasm (CVS) and/or coronary microvascular dysfunction (CMD) [1]. Recent guidelines recommend invasive coronary function testing (CFT) in ANOCA patients to identify a specific CVDys endotype [2]. As a consequence of these recommendations, the pathophysiology of CVDys is becoming more evident [3].

CVDys is a heterogeneous entity and can be caused by structural and/or functional abnormalities of the vasculature. Interestingly, patients with ANOCA are at increased risk for developing HFpEF [3], a condition characterized by structural and electrophysiological remodeling (e.g. QTc interval prolongation [4]). However, the electrophysiological characteristics of ANOCA patients with CVDys are unexplored. We hypothesized that the various CVDys etiologies demonstrate distinct resting electrocardiogram (ECG) patterns as compared to patients without CVDys and analyzed resting ECGs in patients undergoing CFT.

## 2 Methods

**Fig. 1:**
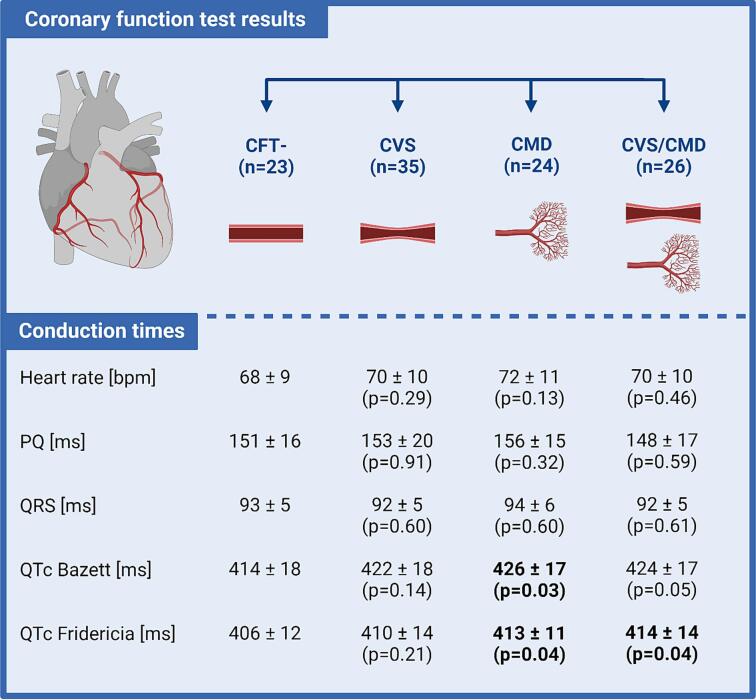
Overview of the ECG characteristics in ANOCA patients diagnosed with and without CVDys. P-values were determined with the unpaired *t*-test and relative to the CFT- group. CFT- = Negative coronary function test result; CVS = Coronary vasospasm; CMD = coronary microvascular dysfunction. Created with BioRender.com.

We analyzed clinical data and rest ECGs of 167 ANOCA patients who participated in the NL-CFT registry and underwent a CFT according to the NL-CFT protocol [5]. Patients were diagnosed with CMD in case of abnormal coronary flow reserve (CFR ≤ 2.0) and/or abnormal index of microvascular resistance (IMR ≥ 25) as measured with the bolus thermodilution method. We defined CVS as the presence of epicardial and/or microvascular vasospasm during acetylcholine spasm provocation testing. To ensure that vasoreactivity is not influenced by medication when performing spasm provocation testing, patients temporarily stopped the intake of long-acting anti-anginal medication and other vasoactive substances 24–48 h before CFT. To improve power, we selected a similar number of patients with only CVS, only CMD, the combined endotype (CVS/CMD), and patients without CVDys based on the CFT result (Fig. 1, upper panel). All patients provided informed consent. We excluded patients (1) without a preprocedural ECG (n = 16), (2) if their ECG did not show sinus rhythm (n = 2), (3) if the ECG showed a complete bundle branch block (n = 8) and (4) in whom no complete CFT was performed (n = 5). Lastly, patients were excluded if they had a history of a percutaneous coronary intervention or a coronary artery bypass graft (n = 28), as we wanted to avoid a possible influence of previous obstructive coronary artery disease on our results. For all included patients, we collected clinical data and a 12-lead pre-procedural resting ECG measured on the day of CFT in PDF (Philips Pagewriter TC50) and raw-format (XML).

The raw ECG data were analyzed using Python (version 3.8.10). We marked the beginning and end of the P-wave, QRS complex and ST segment for all sinus rhythm beats per ECG blinded for CFT outcome. This strategy was used to calculate median conduction times. The QTc interval was corrected for heart rate using Bazett’s formula (QTcB) and Fridericia’s formula (QTcF) [6]. We performed two sub-analyses, one in women and another in patients with CMD. In the patients with CMD we compared the QTc interval between patients with structural CMD [7] (CFR ≤ 2.0 and IMR ≥ 25), functional CMD [7] (CFR ≤ 2.0 and IMR < 25) and patients with only an increased IMR (IMR ≥ 25 and CFR > 2.0). Additionally, we used the heart axis (QRS-axis) calculated by the ECG device. Statistical analyses were performed using R (version 4.2.2). An unpaired *t*-test was used for continuous data with a normal distribution. A Mann-Whitney *U* test was used in case of non-normal distributions. We considered a two-sided P < 0.05 as statistically significant.

## 3 Results

In total, we analyzed 108 patients of whom 95 % were women and the mean age was 58 (±8) years. We included 35 patients with CVS, 24 with CMD and 26 with a combination of both outcomes (CVS/CMD). The group with a negative CFT result (CFT-) and therefore no CVDys were diagnosed as having no cardiac chest pain and consisted of 23 patients.

Table 1 shows the baseline characteristics of all patients, stratified by diagnosis following the CFT. The prevalence of hypertension and a positive family history for cardiovascular disease was higher in all subgroups of patients with CVDys compared to patients without CVDys. Overall, CMD patients had the highest cardiovascular risk profile compared to patients without CVDys, with a higher median BMI and higher prevalence of all cardiovascular risk factors.

**Table 1:** Baseline characteristics stratified by CFT outcome.

	CFT- (n = 23)	CVS (n = 35)	CMD (n = 24)	CVS/CMD (n = 26)
Female sex (%)	91 %	94 %	96 %	100 %
Age [years] (mean ± SD)	57 ± 11	57 ± 8	59 ± 5	59 ± 8
BMI (median [Q1-Q3])	24.1 [21.8–27.4]	26.0 [23.5–28.5]	28.0 [26.5–30.4]	24.6 [22.8–26.7]

*Cardiovascular risk factors*				
Hypertension (%)	6 (26)	18 (51)	18 (75)	14 (54)
Hypercholesteremia (%)	11 (48)	14 (40)	16 (67)	13 (50)
Diabetes Mellitus (%)	1 (4)	3 (9)	4 (17)	1 (4)
Former/current smoker (%)	11 (48)	12 (34)	16 (67)	8 (31)
Positive family history (%)	6 (26)	22 (63)	12 (50)	10 (38)

*Medical history*				
ACS (%)	3 (13)	5 (14)	3 (13)	3 (12)

*Medication use*				
Beta blockers (%)	6 (26)	8 (23)	7 (29)	5 (19)
Calcium channel blockers (%)	13 (57)	27 (77)	19 (79)	19 (73)
Long-acting nitrates (%)	5 (22)	5 (14)	7 (29)	6 (23)
Nicorandil (%)	0 (0)	4 (11)	3 (13)	6 (23)
Antiplatelets (%)	7 (30)	13 (37)	7 (20)	9 (35)
Anti-hypertensives (%)	7 (30)	14 (40)	11 (31)	12 (46)
Cholesterol lowering (%)	14 (61)	12 (34)	16 (67)	15 (58)

*At least one drug per category*				
0 Categories (%)	2 (9)	1 (3)	1 (4)	3 (12)
1 Category (%)	5 (22)	9 (26)	3 (13)	3 (12)
2 Categories (%)	6 (26)	6 (17)	8 (33)	3 (12)
3 Categories (%)	5 (22)	15 (43)	4 (17)	8 (31)
4 Categories (%)	5 (22)	3 (9)	4 (17)	6 (23)
>4 Categories (%)	0 (0)	1 (3)	4 (17)	3 (12)

ACS = Acute coronary syndrome; CFT- = Negative coronary function test result; CVS = Coronary vasospasm; CMD = coronary microvascular dysfunction.

Patients with CVDys were more often prescribed calcium channel blockers and nicorandil compared to patients without CVDys. Furthermore, patients with CVS less often used cholesterol lowering medications as compared to the other groups. Table 1 also shows that in total 6 % of all patients did not use any medication associated with the stated cardiovascular drug categories. On the other hand, 75 % of all patients were prescribed medications for at least two of the cardiovascular drug categories.

We observed a left anterior fascicular block in six patients (CFT-: n = 2, CMD: n = 3, CVS: n = 1). Based on the ECGs showing normal heart axis (−30°–90°, n = 102), no significant differences in heart axis were observed between ANOCA patients without CVDys and CVS, CMD or CVS/CMD (40° ± 30° vs 26° ± 28° (p = 0.09), respectively; 40° ± 30° vs 25° ± 30° (p = 0.12), respectively; 40° ± 30° vs 34° ± 26° (p = 0.46), respectively).

Fig. 1 shows a summary of the main results. Heart rate, PQ interval and QRS duration were comparable between the groups. The QTc interval was statistically significantly longer in the CMD group compared to the group without CVDys, although the difference was small (ΔQTcB = 12 ms, ΔQTcF = 7 ms). Using Fridericia’s formula, the QTc interval was also statistically significantly longer in patients with the combined endotype (CVS/CMD) in comparison to patients without CVDys (ΔQTcF = 8 ms, p = 0.04).

In a sub-analysis in women, the statistically significant difference in QTcF remained between patients without CVDys and patients with CMD (406 ± 12 ms vs 413 ± 11 ms, p = 0.03, respectively) or CVS/CMD (406 ± 12 ms vs 414 ± 14 ms, p = 0.03, respectively). The QTcB between patients without CVDys and CMD was no longer statistically significantly different (415 ± 18 ms vs 426 ± 18 ms, p = 0.06, respectively). Another sub-analysis of the QTc interval in patients with CMD showed a longer QTcB and QTcF interval in patients with structural CMD (n = 4) compared to patients with functional CMD (n = 8) (QTcB: 437 ± 11 vs 430 ± 13 ms (p = 0.40); QTcF: 418 ± 12 vs 415 ± 10 ms (p = 0.63), respectively) and compared to patients with only an increased IMR (n = 12) (QTcB: 437 ± 11 vs 419 ± 20 ms (p = 0.12); QTcF = 418 ± 12 vs 410 ± 12 ms (p = 0.25), respectively), although not significant.

## 4 Discussion

Pre-procedural 12-lead electrocardiograms were comparable between patients with and without CVDys undergoing CFT except for a slightly longer QTc interval in patients with CVDys compared to patients without CVDys. To correct the QT-interval for heart rate, we used two formulas (Bazett’s and Fridericia’s). Bazett’s formula is the most frequently used formula in clinical practice but tends to under-correct the QTc interval at lower heart rates. As the observed heart rates in our cohort were generally low, we also applied Fridericia’s formula which is more suitable for low heart rates [6]. The two formulas gave similar results and showed longer QTc intervals in patients with CVDys, especially in patients with CMD solely or in combination with CVS, as compared to patients without CVDys, although the statistical significance varied between the groups.

The finding of a longer QTc interval in patients with CMD compared to patients without CVDys is in line with previously published studies [8]. Interestingly, our sub-analysis provides a first indication that the QTc interval might particularly be the longest in patients with structural CMD, however the groups were small. QTc interval prolongation can be an effect of many factors, including myocardial ischemia and diabetes mellitus. In our study population the prevalence of diabetes mellitus was low and had therefore little impact. In addition, thyroid hormone levels can influence the QTc interval. Higher levels of free thyroxine (FT4) and the ratio between FT4 and free triiodothyronine were observed in patients with ischemia and non-obstructive coronary artery disease with CMD compared to patients without CMD [9]. Baseline differences might therefore have influenced the results. Unfortunately, we could not investigate differences in thyroid hormone levels in our study population. We recommend considering thyroid hormone levels in future research on the QTc interval in ANOCA patients with CVDys. The longer QTc interval in patients with CVDys compared to patients without CVDys could also be an early sign of electrical remodeling, similar to what is seen in patients with HFpEF. However, the QTc interval in CVDys patients is smaller than seen in HFpEF patients [4] and well within normal values. The clinical relevance of the small difference in QTc interval between patients with CVS, CMD or the combined endotype and patients without CVDys is uncertain, but suggests limited cardiac remodeling in patients with CVDys. This could be seen as advantageous since it possibly provides the opportunity to prevent or reverse cardiac damage and development towards HFpEF.

Notably, the number of males in our study was very low at 5 %. It is therefore uncertain whether these results are generalizable to men with ANOCA. Although current studies indicate that ANOCA is more common in women than in men, the number of men referred for and undergoing coronary function tests is lower than expected, suggesting a potential referral bias [10].

Differences in anti-anginal medication use likely did not influence our results, since intake of long-acting anti-anginal medication and other vasoactive substances was stopped 24–48 h before CFT. It is possible that patients were experiencing complaints at the time the 12-lead rest ECG was obtained. Whether this has affected the measured conduction times in our study is unknown.

This is the first study to examine cardiac rest electrophysiology of ANOCA patients with and without CVDys and takes us one step further in unraveling the pathophysiology of CVDys. However, larger studies are necessary to confirm our findings. Our findings show that the resting ECG of ANOCA patients with CVDys at time of CFT is mostly similar to ANOCA patients without CVDys, even though they have a worse prognosis [11]. To improve prognosis, further understanding of the natural course of CVDys and how it affects cardiac structure and electrophysiology is necessary to discover the time window for optimal effect of specific therapeutic targets.

## 5 Conclusion

Conduction times and heart axis measured on a 12-lead resting ECG were mostly comparable between ANOCA patients without CVDys and patients with invasively diagnosed CVS, CMD or the combined endotype. Patients with CVDys had a slightly longer QTc interval compared to patients without CVDys. The clinical relevance of this difference in QTc interval is uncertain, but suggests limited cardiac remodeling in patients with CVDys undergoing CFT.

## CRediT authorship contribution statement

**Diantha J.M. Schipaanboord:** Writing – review & editing, Writing – original draft, Visualization, Validation, Software, Project administration, Methodology, Investigation, Formal analysis, Data curation, Conceptualization. **Tijn P.J. Jansen:** . **Caïa Crooijmans:** Writing – review & editing, Resources, Methodology, Investigation, Data curation, Conceptualization. **N. Charlotte Onland-Moret:** . **Suzette E. Elias-Smale:** Writing – review & editing, Resources, Investigation. **Aukelien C. Dimitriu-Leen:** Writing – review & editing, Resources, Investigation. **Pim van der Harst:** Writing – review & editing, Supervision. **Tim P. van de Hoef:** Writing – review & editing. **René van Es:** Writing – review & editing, Supervision, Software, Resources, Methodology, Conceptualization. **Peter Damman:** Writing – review & editing, Supervision, Resources, Project administration, Methodology, Investigation, Conceptualization. **Hester M. den Ruijter:** Writing – review & editing, Supervision, Project administration, Methodology, Funding acquisition, Conceptualization.

## Declaration of competing interest

The authors declare that they have no known competing financial interests or personal relationships that could have appeared to influence the work reported in this paper.
